# Prevalence of endometriosis in women undergoing laparoscopic surgery for various gynaecological indications at a Jordanian referral centre: gaining insight into the epidemiology of an important women's health problem

**DOI:** 10.1186/s12905-021-01530-y

**Published:** 2021-10-31

**Authors:** Nadia Muhaidat, Shawqi Saleh, Kamil Fram, Mohammed Nabhan, Nadia Almahallawi, Saif Aldeen Alryalat, Mutasem Elfalah, Mohammed Elfalah

**Affiliations:** 1 Department of Obstetrics and Gynaecology, Faculty of Medicine, The University of Jordan, Queen Rania Street, Amman, 11942 Jordan; 2 Department of Family Medicine, Faculty of Medicine, The University of Jordan, Amman, 11942 Jordan; 3 Department of Ophthalmology, Faculty of Medicine, The University of Jordan, Amman, 11942 Jordan; 4 Faculty of Medicine, The University of Jordan, Amman, 11942 Jordan

**Keywords:** Endometriosis, Laparoscopy, Jordan, Prevalence, Women's health, Infertility, Pelvic pain

## Abstract

**Background:**

Endometriosis is a considerable health challenge for women of reproductive age. Information about its prevalence in the Jordanian population is sparse. The objective of this research was to evaluate the presence of endometriosis in gynaecological patients undergoing laparoscopic surgery for various indications and to correlate the finding of endometriosis with variables, including patient demographics, obstetric history, type, and indication of laparoscopic procedure.

**Methods:**

A retrospective cohort study involving 460 women who underwent different laparoscopic procedures for a variety of indications was conducted in the Department of Obstetrics and Gynaecology in Jordan University Hospital, a tertiary referral hospital in Jordan, between January 2015 and September 2020.

**Results:**

The prevalence of endometriosis in this patient group was higher than that of the general population (13.7% vs. 2.5%), and the mean age at diagnosis (31.9 years) was younger than the general population's age of peak incidence (35–45 years). It was significantly higher in women with lower numbers of pregnancies (*p* = 0.01) and a lower number of Caesarean sections (*p* = 0.05) and in those where the indication for surgery was related to decreased fertility or pelvic pain (*p* = 0.02). Women with high parity or where the surgery's indication suggested normal fertility, such as family planning, were less likely to have endometriosis.

**Conclusion:**

To our knowledge, this is the first Jordanian study to assess the prevalence of endometriosis in women undergoing gynaecological laparoscopy. This study suggests that the epidemiology of endometriosis in this region follows similar trends to what has been previously documented in international literature, while emphasizing the need for further research into this important women's health issue in this part of the world.

## Background

Endometriosis is a benign gynaecological disease characterized by the presence of endometrial tissue in the form of stroma and glands outside the uterus and is associated with a spectrum of clinical presentations, ranging from asymptomatic women to patients suffering debilitating pelvic pain and/or fertility issues [1, 2].

The actual prevalence in the population is difficult to ascertain because the diagnosis is often delayed or overlooked [2], but it is estimated that the condition affects up to 10% of women of reproductive age [3]. A cross-sectional population survey in the United States estimated the prevalence of diagnosed endometriosis to be 6.1% [4]; however, a study conducted in Italy suggests that only 6 out of 10 cases of endometriosis in the general population are diagnosed [5].

Endometriosis in Jordan, a middle-income country in the Middle East, is considered a significant public health issue affecting the quality of life of women of reproductive age [6]. The prevalence according to a community-based survey of women aged 15–55 years by Al-Jefout et al. was found to be 2.5% [6]. In another study, the prevalence of endometriosis in symptomatic young Jordanian women undergoing laparoscopy for refractory chronic pelvic pain was found to be as high as 71.4% [7]. Therefore, there is considerable variation in the prevalence according to the type of population sample under investigation.

The two main manifestations of endometriosis can be categorized into endometriosis-related pain, such as dysmenorrhea, chronic pelvic pain, dyspareunia, dyschezia, and infertility. Symptoms can be significantly disruptive and affect the woman's quality of life. However, 20–25% of women are affected but completely asymptomatic [8–10]. The age of peak incidence was found to be approximately 40 years [11, 12].

There are challenges involved in the process of diagnosing endometriosis. First, the symptoms with which the patient presents may overlap with other conditions, such as adenomyosis, interstitial cystitis and irritable bowel syndrome. Second, the patient may consider her pain to be a normal manifestation of the menstrual cycle, which leads to a delay in seeking medical advice [1].

The gold standard for the diagnosis of endometriosis is direct visualization during laparoscopy [13–15]. According to the appearance of lesions found during surgery, endometriosis can be classified into subtle, typical, cystic, or deep [16]. While this condition is commonly diagnosed during laparoscopy in patients with symptoms of endometriosis [8, 17], it is also found incidentally in patients undergoing laparoscopic surgery for indications other than clinical suspicion of endometriosis. For example, one meta-analysis of asymptomatic patients with clomiphene-resistant polycystic ovary syndrome with endometriosis found a prevalence of surgically confirmed endometriosis of 7.7% [18]. Another study of women asymptomatic for endometriosis undergoing laparoscopy for other indications by Rawson found a prevalence of endometriosis of as much as 43.5% and demonstrated that subtle endometriotic changes were commonly found during laparoscopy in women who denied typical symptoms of the condition and reported normal fertility [19].

Several other factors have been found to be associated with the presence of endometriosis. A meta-analysis by Yong and Weiyuan suggested that a higher body mass index may be associated with a lower risk of endometriosis [20]. This finding agrees with Ferrero et al. who demonstrated that women with endometriosis have lower body mass indices and are less frequently obese than women who do not suffer from endometriosis [21]. Furthermore, Matalliotakis et al. found that women with endometriosis had a lower body weight and fewer prior pregnancies, elective abortions and ectopic pregnancies than women seeking care for infertility who did not have endometriosis [22]. An inverse relationship between gravidity and endometriosis was also found within a subgroup of subjects who had diagnostic laparoscopy [23].

This study aims to elaborate more on the prevalence of endometriosis in Jordanian women undergoing laparoscopy for different indications.

## Methods

### Study type and objective

This is a retrospective cohort study involving a sample of 460 women who underwent different laparoscopic procedures for a variety of indications in the Department of Obstetrics and Gynaecology in Jordan University Hospital, a tertiary referral hospital in Jordan, between January 2015 and September 2020.

The objective of this research was to determine the prevalence of endometriosis diagnosed during laparoscopy and to correlate the finding of endometriosis with several variables, including age, body mass index (BMI), obstetric history, type of laparoscopic procedure, and indication for surgery.

### Inclusion and exclusion criteria

The inclusion criteria for the study were as follows: (1) female patient of reproductive age (16–50 years of age) and (2) undergoing gynaecological laparoscopy between January 2015 and September 2020. Patients were excluded from the study if they were under 16 years or over 50 years of age or if they were already diagnosed or treated for endometriosis prior to the index surgery.

### Data collection

Patients undergoing gynaecological laparoscopy were identified from the clinical and theatre records. A total of 460 patients qualified for inclusion. Data collection was performed by retrospectively reviewing the electronic medical records of the patients. The diagnosis of endometriosis was made by one of six consultants with longstanding experience in laparoscopic surgery and the diagnosis of endometriosis. Positive cases were identified based on documentation of the presence of endometriotic lesions (subtle, typical, cystic, or deep) in the electronic operative notes of the laparoscopic surgery. Where histological examination of tissue samples was available, this was correlated with clinical findings, with 100% agreement between clinical and histological diagnoses.

### Ethical approval

Ethical approval was granted by the Institutional Review Board of Jordan University Hospital prior to commencing data collection. Reference number 1012021/3168.

### Statistical analysis

We used SPSS version 26.0 (Chicago, USA) in our analysis. Mean (± standard deviation) was used to describe continuous variables (e.g., age and BMI). Count (frequency) was used to describe other nominal variables.

We performed a chi-square test followed by Z-test for proportions to analyse the difference in frequency for procedure, indication, parity, miscarriages, ectopic pregnancies, and Caesarean sections between patients with and without endometriosis. We used an independent sample t-test to analyse the mean difference between measurements (e.g., age and BMI) and the presence of endometriosis. We adopted a p value of 0.05 as a significance threshold.

We performed a logistic regression analysis to find factors associated with endometriosis on multivariate level. We included variables with *p* value < 0.1 on univariate analysis. We reported model classification accuracy and Nagelkerke R Square. We reported odds ratio and their 95% confidence interval (CI) for variables with p value of 0.05.

## Results

### Study sample demographics

A total of 460 patients who underwent gynaecological laparoscopic surgery between 2015 and 2020 were included in this study, with a mean age of 33.09 (± 7.45) years (SD). Characteristics of the included sample, including age; BMI; parity; and number of miscarriages, ectopic pregnancies, and Caesarean sections, are illustrated in Table 1.

**Table 1:** Main demographic characteristics of patients included in the study sample

Characteristics	Minimum	Maximum	Mean	SD
Age	16	50	33.09	7.454
BMI	17	46	26.84	4.658
Parity	0	8	1.60	1.933
Miscarriages	0	16	0.59	1.620
Ectopic pregnancies	0	2	0.08	0.296
Caesarean sections	0	5	0.51	1.034

### Overall prevalence of endometriosis

The overall prevalence of endometriosis in all patients was 13.7% (Table 2). Diagnostic laparoscopy was the most common procedure performed on 251 (54.6%) patients. Infertility was the most common indication in 177 (38.5%) patients. Diagnosis was made by the surgeon, identifying features of endometriosis. According to the description of lesions provided in the patients' records, the type of endometriosis was determined (subtle, typical, cystic or deep) by the surgeon during the procedure. Histopathological examination of suspected endometriotic tissue samples was available for 13 patients (21% of those with clinical evidence of endometriosis), and where available, there was 100% agreement with clinical findings. These were patients who had prior to surgery consented to the excision of the cystic lesion or oophorectomy and where an intraoperative diagnosis of ovarian endometrioma was made based on gross appearance. This was then confirmed by histopathology for all cases. Histopathological examination was performed by one of two pathologists with special interest and expertise in gynaecological diseases.

**Table 2:** Numbers and percentages of patients with and without evidence of endometriosis during laparoscopy

Evidence of endometriosis and its subtypes	Number	Percentage (%)
No	397	86.3
Yes	63	13.7
Subtle	16	25.4*
Typical	26	41.3*
Cystic	17	27.0*
Deep	4	6.3*

*Represented as a percentage of total cases with endometriosis

Eighteen (28.6%) patients had some form of surgical treatment (endometrioma resection, ablation of endometriotic lesions, or adhesiolysis at the time of primary operation), as there was previous suspicion of the presence of pathology; hence, patients consented for further surgical management. For a further 10 (15.9%) patients, they were recommended to undergo further surgery to treat endometriosis as a second step procedure after obtaining proper consent and preparing the patient. In the remaining patients, the finding of endometriosis was not associated with symptoms related to the disease; it was therefore deemed incidental, not requiring surgical treatment at that stage.

Tables 3 and 4 contain the types of laparoscopic surgeries that were performed on the patient sample of this study and their indications.

**Table 3:** Types of laparoscopic surgeries performed on 460 patients

Procedure	Number	Percentage (%)
Diagnostic laparoscopy	251	54.6
Laparoscopic ovarian cystectomy	121	26.3
Laparoscopic salpingectomy/salpingostomy	48	10.4
Tubal ligation, bilateral or unilateral	37	8.0
Laparoscopic oophorectomy	1	0.2
Laparoscopically assisted vaginal hysterectomy	2	0.4

**Table 4:** Indications for laparoscopic surgeries performed on 460 patients

Indication for laparoscopy	Number	Percentage (%)
Suspected uterine perforation	31	6.7
Infertility, primary or secondary	177	38.5
Ovarian cyst accident	125	27.2
Ectopic pregnancy	47	10.2
Family planning	37	8.0
Chronic pelvic pain	16	3.5
Recurrent pregnancy loss	6	1.3
Other	21	4.6

Prevalence of endometriosis according to indication for laparoscopy

Upon comparing the frequency of endometriosis among different groups, we found a statistically significant difference in the prevalence of endometriosis in patients undergoing laparoscopy according to indication (*p* = 0.020), as detailed in Table 5. On post hoc Z-test for proportions, the difference was most significant for family planning, where all 37 (100%) patients undergoing laparoscopies for family planning indication were negative, and for chronic pelvic pain, where 5 (31.3%) endoscopies were positive. The condition was most commonly found when the indication for laparoscopy was chronic pelvic pain (31.3%), followed by infertility (16.9%), recurrent pregnancy loss (16.7%), ovarian cyst (16%), diagnosis after suspected perforation (9.7%), and ectopic pregnancy (4.3%). No cases of endometriosis were identified in those undergoing laparoscopies for family planning purposes, such as tubal ligation.

**Table 5:** Numbers and percentages of patients with versus without evidence of endometriosis according to the indication for laparoscopy

Indication for laparoscopy	No endometriosis number (%)	Endometriosis number (%)	*p* value
Suspected uterine perforation	28 (90.3)	3 (9.7)	0.020
Infertility, primary or secondary	147 (83.1)	30 (16.9)	
Ovarian cyst accident	105 (84%)	20 (16%)	
Ectopic pregnancy	45 (95.7)	2 (4.3)	
Family planning	37 (100.0)	0 (0.0)	
Chronic pelvic pain	11 (68.8)	5 (31.3)	
Recurrent pregnancy loss	5 (83.3)	1 (16.7)	
Other	19 (90.5)	2 (9.5)	

### Prevalence of endometriosis according to type of laparoscopic procedure

Upon comparing different procedures with the presence of endometriosis, we did not find a significant difference (*p* = 0.059). Table 6 details the frequencies. In all, 50% of those undergoing laparoscopically assisted vaginal hysterectomy had endometriosis; however, as only two patients were included in this group, it may not be very representative. Endometriosis was found in 15.9% of patients who underwent diagnostic laparoscopy, followed by those who underwent laparoscopic ovarian cystectomy (14.9%). Less frequently, the finding of endometriosis was associated with laparoscopic salpingectomy or salpingostomy (8.3%), and no cases of endometriosis were identified among patients who had laparoscopic oophorectomy or tubal ligation.

**Table 6:** Numbers and percentages of patients with versus without evidence of endometriosis according to the type of laparoscopic procedure

Procedure	No endometriosis number (%)	Endometriosis number (%)	*p* value
Diagnostic laparoscopy	211 (84.1)	40 (15.9)	0.059
Laparoscopic ovarian cystectomy	103 (85.1)	18 (14.9)	
Laparoscopic salpingectomy/salpingostomy	44 (91.7)	4 (8.3)	
Tubal ligation, bilateral or unilateral	37 (100.0)	0 (0.0)	
Laparoscopic oophorectomy	1 (100.0)	0 (0.0)	
Laparoscopically assisted vaginal hysterectomy	1 (50.0)	1 (50.0)	

### Prevalence of endometriosis according to age, BMI, parity, number of miscarriages, ectopic pregnancies and caesarean sections

As shown in Table 7, the mean age of patients with endometriosis was 31.97 (SD 6.55) years, while it was 33.27 (SD 7.58) years for those without endometriosis. This difference was not statistically significant (*p* = 0.155).

**Table 7:** Numbers and means of patients with versus without evidence of endometriosis according to the type of laparoscopic procedure

Procedure	No endometriosis Mean ± SD	Endometriosis Mean ± SD	*p* value
BMI	27.0 ± 4.7	26.0 ± 4.3	0.130
Age	33.27 ± 7.58	31.97 ± 6.55	0.155

In this study population, no significant association was found between the patient's body mass index (BMI) and the presence of endometriosis (*p* = 0.13). The mean BMI for patients with a positive finding of endometriosis was 26.4 (SD 4.3) kg/m^2^, whereas it was 27.0 (SD 4.7) kg/m^2^ for those without endometriosis (Table 7).

Out of the 460 participants, previous obstetric history data were available for 436 patients. Endometriosis was significantly higher in patients with a lower number of parities (*p* = 0.008) and Caesarean sections (*p* = 0.035) (Table 8). Regarding parity, endometriosis was found in patients with parities up to 4, with no cases of endometriosis found in grand multipara. The prevalence in nulliparous women was 19%. It was 19.7% in women with a parity of one, 5.3% in women with a parity of two, 19.0% in women with a parity of 3, and 2.4% in women with a parity of 4.

**Table 8:** Numbers and percentages of patients with versus without evidence of endometriosis according to parity and number of miscarriages, ectopic pregnancies, and Caesarean sections

	Number	No endometriosis number (%)	Endometriosis number (%)	*p* value
Parity	0	153 (81.0)	36 (19.0)	0.008
	1	57 (80.3)	14 (19.7)	
	2	54 (94.7)	3 (5.3)	
	3	34 (81.0)	8 (19.0)	
	4	39 (97.5)	1 (2.5)	
	5	17 (100.0)	0 (0.0)	
	6	7 (100.0)	0 (0.0)	
	7	6 (100.0)	0 (0.0)	
	8	7 (100.0)	0 (0.0)	
Miscarriages	0	267 (83.4)	53 (16.6)	0.499
	1	53 (88.3)	7 (11.7)	
	2	27 (96.4)	1 (3.6)	
	3	11 (91.7)	1 (8.3)	
	4	9 (100.0)	0 (0.0)	
	5	1 (100.0)	0 (0.0)	
	6	3 (100.0)	0 (0.0)	
	15	1 (100.0)	0 (0.0)	
	16	2 (100.0)	0 (0.0)	
Ectopic pregnancies	0	343 (84.9)	61 (15.1)	0.051
	1	29 (100.0)	0 (0.0)	
	2	2 (66.7)	1 (33.3)	
Caesarean sections	0	266 (82.6)	56 (17.4)	0.035
	1	47 (90.4)	5 (9.6)	
	2	34 (100.0)	0 (0.0)	
	3	15 (93.8)	1 (6.3)	
	4	6 (100.0)	0 (0.0)	
	5	6 (100.0)	0 (0.0)	

The prevalence of endometriosis was highest in women with no previous Caesarean sections (17.4%), with those who had from one to five Caesarean sections having a prevalence less than the overall study population.

The prevalence of endometriosis was highest in those who had not suffered any miscarriages (16.6%), followed by those who had one (11.7%), three (8.3%), or two (3.6%) miscarriages. None of the patients suffering four or more miscarriages were found to have endometriosis. In relation to ectopic pregnancy, the prevalence was 15.1% for those with no previous ectopic pregnancy and 33.3% for those with two previous ectopic pregnancies, whereas none of the patients with a history of one ectopic pregnancy were found to have the condition. The association between endometriosis and the number of ectopic pregnancies and miscarriages was not found to be statistically significant.

We performed a logistic regression analysis to find factors associated with endometriosis on multivariate level. We included variables with *p* value < 0.1 on univariate analysis. The model has a classification accuracy of 86.4% with Nagelkerke R Square of 0.094. Parity and cesarean section were significantly associated with endometriosis (*p* values of 0.036 and 0.046, respectively). Their odds ratios were 0.8 (95% CI 0.65–0.9) and 0.54 (95% CI 0.3–0.9), respectively.

## Discussion

The need to investigate the epidemiology of endometriosis was recognized a long time ago [24]. The true prevalence of endometriosis within any given population is difficult to determine due to a number of factors. The estimated prevalence varies according to geographical area, patient group under investigation, presence versus absence of symptoms, and method of diagnosis. Furthermore, a significant number of cases may never be diagnosed, as medical advice is not sought by the patient due to lack of symptoms or normalization of existing symptoms [1–7]. As diagnosis is typically only confirmed after laparoscopy, data involving asymptomatic patients are likely deficient [25].

Furthermore, epidemiological studies of endometriosis face a number of methodological challenges, such as disease definition, selection bias, and challenges associated with performing cohort or case control studies [16, 26].

This study found evidence of endometriosis in 13% of the 480 women undergoing gynaecological laparoscopic surgery between 2015 and 2020. This is considered higher than the prevalence in the general population (2.5%) [6]. For some of these patients, laparoscopy was indicated by conditions that are potentially related to endometriosis, such as infertility (37.1%) and chronic pelvic pain (3.3%), whereas for others, the indication did not bear an obvious connection to this condition, such as family planning (7.7%).

The mean age of women who were found to have endometriosis was 31.97 (SD 6.55) years. This is younger than the age of peak prevalence of endometriosis reported in previous studies by Eisenberg et al. and Abbas et al. who stated a peak prevalence in the late thirties and early forties [2, 27]. No statistically significant difference was found in the average age or BMI of patients diagnosed with endometriosis and those who were not. This contrasts with previous studies that demonstrated that women with endometriosis tend to have a lower body mass index than the general population [21–23].

There was a statistically significant correlation between the indication for laparoscopy and the percentage of patients who were found to have endometriosis (*p* = 0.02). This was highest in those where the indication was chronic pelvic pain (31.2%) and infertility (16.9%). Patients who underwent laparoscopy for recurrent pregnancy loss and ovarian cyst accidents were also found to have endometriosis more frequently than the overall 13%. Other indication groups had a lower prevalence of endometriosis, with those undergoing laparoscopies for family planning purposes noticeably having a 0% prevalence of endometriosis. This compares to a study by Mahmood and Templeton who evaluated the prevalence of endometriosis in premenopausal Caucasian women undergoing laparoscopy according to indication and found that it was 21% for those who underwent laparoscopy to investigate infertility, 15% in those with chronic abdominal pain, and 6% in women undergoing laparoscopic sterilization [28].

There was no significant difference in the prevalence of endometriosis when correlated with the type of laparoscopic surgery. Nevertheless, it should be noted that no cases of endometriosis were found in the patient subgroups undergoing laparoscopy for tubal ligation or oophorectomy.

The prevalence of endometriosis was significantly higher in patients with lower parities and numbers of Caesarean sections. This could be explained by the known association between endometriosis and infertility [10, 29].

No significant association was found between the prevalence of endometriosis and the number of previous miscarriages or ectopic pregnancies, but a noticeably high percentage (33.3%) of women who had 2 previous ectopic pregnancies were found to have endometriosis. This agrees with previous publications that suggest an association between the occurrence of ectopic pregnancy and the presence of endometriosis [30, 31].

## Conclusion

There is to date a paucity of information regarding the true prevalence of endometriosis in Jordan. The numbers vary greatly according to the type of population examined in any given study. To our knowledge, this is the first Jordanian study to assess the prevalence of endometriosis in women undergoing gynaecological laparoscopy. These patients comprise a heterogeneous group regarding the indication that prompted the procedure.

The overall presence of endometriosis upon laparoscopy in this study (13%) was found to be higher than the global prevalence of this condition; however, the study sample does not represent the general population, in which the prevalence is estimated to be 2.5%, but rather a group of patients with some gynaecological complaint that requires laparoscopic diagnosis or treatment. We also found that, when endometriosis prevalence is investigated in a population sample undergoing laparoscopic gynaecological surgery, the mean age of affected patients is lower than the general age of peak incidence for this condition.

There is significant variation in the prevalence of endometriosis in these patients according to several factors. The findings of this study suggest that the finding of endometriosis in Jordanian women undergoing gynaecological laparoscopy is more commonly encountered in women with lower numbers of pregnancies and in those where the indication for surgery was related to decreased fertility or pelvic pain. Women with high parities or where the surgery's indication suggested normal fertility, such as family planning, were less likely to have endometriosis.

Patient age and BMI did not seem to have a significant association with endometriosis in this study population.

Endometriosis is a significant public health concern in Jordan, as it is worldwide, but its epidemiology in this region is still poorly understood. We hope that this research will add to our awareness of the prevalence of this disease in the Middle East and prompt further research in this field.

This research was limited by the fact that it was a retrospective single-centre study performed on a relatively small sample of patients. Due to the retrospective nature of the research, histological diagnosis was not available for all cases. Biopsy is not routinely performed in our centre to diagnose cases of endometriosis, and as this study was retrospective, only data available in the patients' records could be extracted and analysed. Additional surgical procedures would require informed consent, and in many cases, the finding of endometriosis was incidental in a surgical operation with an unrelated indication. However, in those where it was available, there was a 100% correlation with clinical findings. Further large-scale multicentre prospective studies are required to deepen our understanding of endometriosis in Jordan and the Middle East.

## Data Availability

The dataset used and/or analysed during the current study is available from the corresponding author on reasonable request.

## Abbreviations

BMI: Body mass index
SPSS: Statistical package for the social sciences
SD: Standard deviation
